# Current Perspectives on Balloon Pulmonary Angioplasty for Chronic Thromboembolic Pulmonary Hypertension

**DOI:** 10.3390/jcm15010051

**Published:** 2025-12-21

**Authors:** Hyungdon Kook, Woohyeun Kim, Ran Heo, Kyunam Kim, Seung-Jin Yoo, Hyunsoo Kim, Dong Won Park, Young-Hyo Lim

**Affiliations:** 1 Division of Cardiology, Department of Internal Medicine, College of Medicine, Hanyang University, 222 Wangsimni-ro, Seongdong-gu, Seoul 04763, Republic of Korea; cvkook@hanyang.ac.kr (H.K.); coincidence1@naver.com (W.K.);; 2 Department of Anesthesiology and Pain Medicine, College of Medicine, Hanyang University, Seoul 04763, Republic of Korea; knkim9@hanyang.ac.kr; 3 Department of Radiology, College of Medicine, Hanyang University, Seoul 04763, Republic of Korea; 4 Department of Thoracic and Cardiovascular Surgery, College of Medicine, Hanyang University, Seoul 04763, Republic of Korea; 5 Division of Pulmonary Medicine and Allergy, Department of Internal Medicine, College of Medicine, Hanyang University, Seoul 04763, Republic of Korea; dongwonpark@hanyang.ac.kr

**Keywords:** chronic thromboembolic pulmonary hypertension, balloon pulmonary angioplasty, pulmonary endarterectomy, riociguat, pulmonary hypertension

## Abstract

Balloon pulmonary angioplasty (BPA) has become an established treatment modality for patients with inoperable chronic thromboembolic pulmonary hypertension (CTEPH), particularly in those with distal pulmonary artery lesions or significant comorbidities precluding pulmonary endarterectomy. BPA provides significant improvement in pulmonary hemodynamics, right ventricular function, exercise tolerance, and quality of life. Recent randomized controlled trials, including the RACE and MR-BPA trials, have demonstrated that BPA results in greater reduction in pulmonary vascular resistance and mean pulmonary arterial pressure compared to riociguat, although with a higher incidence of procedure-related complications. Ancillary follow-up data further suggest that a sequential strategy combining medical therapy and BPA may optimize outcomes and reduce adverse events. Advances in procedural techniques, imaging guidance, and patient selection have substantially improved the safety profile of BPA. International registries and expert consensus guidelines now support its incorporation into the multimodal management of CTEPH. This review synthesizes current evidence on the efficacy, safety, and practical aspects of BPA, while highlighting ongoing challenges, including long-term outcome data, standardization of treatment endpoints, and the role of combination therapy. BPA is poised to play an increasingly central role in personalized care strategies for CTEPH.

## 1. Introduction

Chronic thromboembolic pulmonary hypertension (CTEPH) is a progressive form of pulmonary hypertension caused by unresolved, organized thrombi in pulmonary arteries and secondary arteriopathy that leads to elevated pulmonary vascular resistance (PVR) and right ventricular failure [1]. Epidemiological studies estimate the prevalence of CTEPH at roughly 26–38 cases per million and an incidence of 3–5 cases per million adults per year, although precise numbers are difficult to establish due to under-recognition and misdiagnosis [2,3]. CTEPH develops in approximately 3–4% of patients after an episode of acute pulmonary embolism despite adequate anticoagulation [4]. The gold standard treatment is pulmonary endarterectomy (PEA), an open surgical procedure performed under deep hypothermic circulatory arrest that removes organized thrombotic material from main and lobar pulmonary arteries. When successful, PEA is curative; however, up to one-third of patients are deemed inoperable because thrombi are located in distal segmental or subsegmental branches or because comorbidities, advanced age or patient preference preclude surgery [5]. Moreover, residual or recurrent pulmonary hypertension persists in approximately 30% of patients after PEA [6]. Pharmacological therapy targeting the microvascular component—including endothelin receptor antagonists, phosphodiesterase-5 inhibitors and prostacyclin analogs—provides modest improvement but does not relieve mechanical obstruction [7,8,9,10]. The soluble guanylate cyclase stimulator riociguat is currently the only medication specifically approved for CTEPH [11]; it improves exercise capacity and hemodynamics but leaves a large unmet need [12]. Balloon pulmonary angioplasty (BPA) represents a therapeutic approach that contributes to narrowing this gap.

## 2. Pathophysiology of CTEPH

CTEPH arises when organized thrombi adhere to the pulmonary arterial wall and fail to undergo normal fibrinolysis. The chronic thrombus comprises collagen, elastin and recanalized channels lined by dysfunctional endothelium, leading to fixed mechanical obstruction [13]. Increased shear stress and hypoxemia in unobstructed regions trigger a secondary arteriopathy characterized by intimal fibrosis, smooth-muscle proliferation and microvascular rarefaction, similar to changes seen in pulmonary arterial hypertension [14,15]. The combined mechanical and microvascular components elevate PVR and mean pulmonary arterial pressure (mPAP), imposing a sustained afterload on the right ventricle (RV). Over time, the RV hypertrophies and dilates, with deterioration in systolic and diastolic function eventually leading to right-heart failure and even death if untreated. Patients typically present with progressive dyspnea, exercise intolerance and signs of right-heart dysfunction; some develop hemoptysis, syncope or thromboembolic events. Early diagnosis is challenging because symptoms overlap with other forms of pulmonary hypertension and chronic lung disease [16]. Ventilation–perfusion (V/Q) scintigraphy is highly sensitive for detecting perfusion defects and remains the recommended screening test; mismatched segmental defects suggest chronic thromboembolic disease [17,18]. Computed tomography pulmonary angiography (CTPA) provides high-resolution images of the pulmonary arteries, revealing webs, bands, abrupt narrowing and eccentric thrombus [19]. Magnetic resonance pulmonary angiography and digital subtraction angiography further delineate lesion anatomy [1]. The recognition that surgical accessibility is limited to proximal lesions and that distal perfusion can be restored by catheter-based techniques provided the rationale for developing BPA.

## 3. Historical Development of BPA

The earliest attempts at percutaneous dilation of chronically occluded pulmonary arteries date to the late 1980s, but it was not until the early 2000s that the procedure attracted wider attention [20]. Feinstein and colleagues reported a seminal series in 2001, describing 18 patients who underwent BPA for inoperable CTEPH [21]. They achieved significant reductions in mPAP and improvements in New York Heart Association functional class and 6 min walk distance (6MWD) but encountered a high incidence of reperfusion pulmonary injury (RPI) of 61%. However, Japanese physicians who continued to refine the BPA technique recognized that the use of oversized balloons and the treatment of multiple pulmonary artery segments within a single session contributed significantly to procedural complications. Mizoguchi and colleagues developed a refined BPA strategy that began with the use of small-diameter balloons and progressively employed larger sizes in subsequent sessions, while limiting the number of segments treated per procedure. They also introduced intravascular ultrasound (IVUS) and pressure wire measurements to guide balloon sizing and enhance procedural safety [22]. This refined strategy substantially reduced serious adverse events. With improved safety, Japanese centers reported sustained hemodynamic improvement and survival rates, reigniting interest in BPA globally.

## 4. Patient Selection and Multidisciplinary Team Evaluation

Optimal outcomes with BPA depend on meticulous patient selection and multidisciplinary evaluation. According to the ESC/ERS guidelines, BPA is indicated for patients with CTEPH deemed inoperable due to predominantly distal lesions or prohibitive surgical risk and for those with persistent or recurrent pulmonary hypertension after PEA [19]. Candidates should have World Health Organization (WHO) functional class II–IV symptoms despite anticoagulation and, ideally, undergo at least three months of targeted medical therapy to optimize hemodynamics [19,23]. High baseline PVR, elevated mPAP, and poor exercise capacity increase the risk of complications and should be carefully weighed [24,25]. Patients with chronic thromboembolic disease without resting pulmonary hypertension (CTEPD) may benefit symptomatically from BPA, but long-term hemodynamic outcomes remain uncertain.

A comprehensive evaluation by a dedicated CTEPH team improves decision making [26]. Multidisciplinary teams include pulmonologists, cardiologists, radiologists, cardiothoracic surgeons, and anesthesiologists. Joint review of imaging, hemodynamics, functional status, and patient preferences supports well-balanced and tailored therapeutic planning. Assessment includes echocardiography, pulmonary function tests, right-heart catheterization, V/Q scintigraphy, CTPA and, in selected cases, cardiopulmonary exercise testing. Selective pulmonary angiography with pressure wire measurements helps confirm lesion accessibility and gauge distal vessel resistance. Left-heart disease, chronic thromboembolic disease due to antiphospholipid syndrome or myeloproliferative disorders, chronic renal insufficiency or severe parenchymal lung disease may influence the treatment strategy. Comorbid left-heart disease is important because elevated left ventricular filling pressure increases the risk of pulmonary edema during BPA and independently predicts mortality in CTEPH [27]. Careful assessment of left-sided structure and function, including echocardiography and, when indicated, direct measurement of left ventricular filling pressure, is therefore helpful before committing a patient to BPA, and in such patients smaller balloons, fewer segments per session and meticulous fluid management are crucial. Severe parenchymal lung disease such as advanced chronic obstructive pulmonary disease or interstitial lung disease also modifies the risk–benefit profile of BPA. These patients have limited ventilatory reserve and are more prone to hypoxemia and reperfusion injury, and imaging often reveals a mixture of vascular and parenchymal causes of perfusion defects. In this context, V/Q scintigraphy, dual-energy CT perfusion maps and detailed high-resolution CT are useful for distinguishing predominantly vascular lesions that may benefit from BPA from diffuse parenchymal abnormalities in which improvement is likely to be modest. When BPA is pursued in such patients, it is generally advisable to adopt a conservative strategy with staged treatment of a small number of segments per session and close monitoring of gas exchange. Patients considered for BPA should ideally be referred to high-volume centers with extensive experience, given the steep learning curve and the correlation between procedural volume and outcomes [28].

## 5. Lesion Classification 

**Figure 1:**
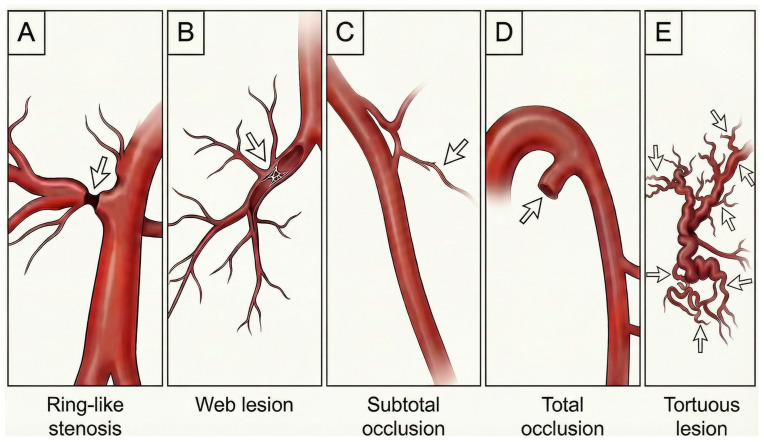
Schematic illustrations demonstrate typical vascular lesion types encountered in CTEPH. (**A**) Ring-like stenosis, characterized by a circumferential, concentric narrowing of the vessel lumen. (**B**) Web lesion, showing intraluminal fibrous bands with complex, mesh-like structures partially obstructing blood flow. (**C**) Subtotal occlusion, in which severe luminal narrowing persists with minimal residual distal flow. (**D**) Total occlusion, often presenting as a pouch-like or ostial obstruction with complete interruption of antegrade flow. (**E**) Tortuous lesion, involving markedly distorted and convoluted distal vessels, typically affecting small-caliber pulmonary arteries.

The success of BPA depends not only on patient selection but also on meticulous lesion assessment and procedural planning. The 2023 ESC consensus statement introduced lesion types A through E, categorized according to angiographic morphology and hemodynamic characteristics (Figure 1) [29]. Type A lesions are ring-like stenoses that create a concentric narrowing resembling a band around the vessel; these are typically addressed first because the risk of vessel injury is low. Type B web lesions appear as hazy or abruptly narrowed opacities that may take various forms, including complex webs or slit-like patterns, and they generally respond well to low-pressure balloon dilation. Type C and Type D lesions represent occlusions: Type C consists of tapered subtotal occlusions that appear nearly complete but still demonstrate faint continuous or intermittent distal perfusion, whereas Type D lesions are total occlusions presenting as pouch-like or ostial blockages. Type E lesions are tortuous webs or occlusions located in small, highly twisted peripheral vessels beyond the subsegmental arteries and are often accompanied by cotton-wool–like capillary staining. Because Type D and Type E lesions carry a higher risk of perforation and reperfusion injury, they are typically reserved for later treatment sessions or may be avoided altogether. Comprehensive angiographic evaluation with IVUS or optical coherence tomography (OCT) may help classify these lesions. A structured approach to lesion classification allows operators to treat safer lesions first, build experience and gradually tackle more complex anatomy.

## 6. Multimodality Imaging and Functional Assessment in BPA

Imaging advances have enhanced procedural planning and execution. V/Q scintigraphy remains invaluable for initial screening and follow-up, as improvements in perfusion distribution after BPA correlate with clinical outcomes [30,31]. Echocardiography remains central to assessing RV size, function and estimated systolic pulmonary arterial pressure, and is used intra-procedurally to monitor for pericardial effusion or significant valvular abnormalities. CTPA provides high-resolution images for lesion identification and measurement of RV and LV size and function. Haramati et al. described characteristic CT features of chronic thromboembolic disease, including eccentric intraluminal filling defects, webs, bands and tortuous collateral vessels [32]. Recognition of these patterns is essential for distinguishing CTEPH from other causes of pulmonary hypertension and for planning interventions. A recent study introduced a fully automated Bayesian analysis of CTPA to quantify perfusion changes, demonstrating strong correlation with hemodynamic and clinical parameters and potential utility in patient selection and monitoring [33]. Dual-energy CT provides iodine maps that quantify regional blood volume, allowing objective evaluation of perfusion [17,34]. Several studies have shown that CT-derived perfusion metrics, particularly dual-energy CT–based lung perfused blood volume, demonstrate moderate to strong correlations with mPAP, PVR, cardiac output, and 6MWD [30,35,36]. These observations suggest that regional perfusion abnormalities captured by CT imaging reflect the underlying pulmonary vascular load and its functional consequences. The application of artificial intelligence to dual-energy CT for assessing pulmonary perfusion blood volume represents an emerging and actively explored field of research [37]. MRI with four-dimensional flow sequences can visualize pulmonary arterial flow patterns and quantify vortex formation and wall shear stress, potentially predicting areas prone to thrombosis or vascular injury [38,39,40].

During the procedure, digital subtraction angiography is the gold-standard imaging modality. IVUS offers cross-sectional imaging, whereas OCT provides high-resolution luminal images ideal for differentiating webs, bands and wall thickening [22]. Pressure wire technology allows measurement of distal pressure and can inform decisions about lesion severity and adequacy of dilatation [41]. Cone-beam CT can create patient-specific roadmaps [13,29]. A comprehensive interventional imaging roadmap—encompassing echocardiography, V/Q scintigraphy, CTPA, selective pulmonary angiography, IVUS, and OCT—is essential for procedural success and for minimizing complications [42]. The integration of artificial intelligence to automatically segment pulmonary arteries, identify lesions and recommend optimal treatment strategies is a promising area of research [43].

Functional assessment is a cornerstone of both patient selection and follow-up in BPA programs. The 6MWD is simple and widely available; improvement in 6MWD after BPA parallels the reduction in PVR and mPAP and has been associated with better long-term outcomes in registry and cohort studies. Moreover, patients with severely reduced baseline 6MWD often derive large absolute gains but may also be at higher risk of periprocedural complications, underscoring its dual role as a prognostic and safety marker [25,44].

Cardiopulmonary exercise testing (CPET) provides more granular insight into the mechanisms of exercise limitation. In CTEPH, a characteristic pattern of reduced peak oxygen uptake, increased ventilatory equivalent for carbon dioxide and preserved or only mildly reduced ventilatory capacity indicates a predominantly circulatory limitation and has been shown to predict clinical outcomes beyond resting hemodynamics [45,46]. In patients with CTEPD, symptoms may be disproportionate to resting hemodynamic findings, making functional assessment central to clinical decision making. CPET can uncover a pulmonary vascular pattern of exercise limitation in this population, characterized by ventilatory inefficiency with an increased VE/VCO_2_ slope and impaired gas exchange, even when resting echocardiography and right-heart catheterization do not demonstrate overt pulmonary hypertension [47]. CPET therefore offers complementary information for selecting candidates for BPA, for distinguishing deconditioning or parenchymal lung disease from pulmonary vascular limitation and for quantifying functional recovery after successful revascularization.

High-risk features identified during the initial evaluation and follow-up help to refine therapeutic strategy and to anticipate complications. Table 1 summarizes how the main diagnostic modalities contribute to risk stratification in patients considered for BPA and how they are used during long-term surveillance.

## 7. Procedural Technique of BPA 

**Figure 2:**
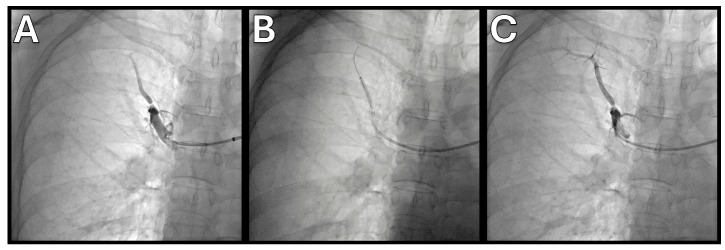
Representative pre- and post-BPA angiographic images. (**A**) Baseline right upper lobe A1 ring-like stenosis lesion. (**B**) Balloon angioplasty using 2.5 mm × 20 mm semi-compliant balloon with nominal pressure, 6 atm. (**C**) Successful dilation of the lesion after BPA.

Current BPA procedure involves staged angioplasty sessions with meticulous attention to technique and imaging. Access is typically obtained via the femoral (preferred) or internal jugular vein, using a long sheath within the short sheath. And a multipurpose guiding catheter (MPA-1) or Judkins right catheter is advanced into the targeted pulmonary artery [48]. Operators often use 0.014 inch atraumatic workhorse guidewires to navigate through the lesion under fluoroscopic guidance. IVUS or OCT may be employed to assess lumen diameter, wall thickness and plaque morphology, enabling appropriate balloon selection [29,42]. Pressure wire measurements across the lesion help determine the residual gradient [49]. Balloons of 2–4 mm diameter are commonly used (Figure 2); oversizing may increase the risk of vessel injury. Balloon inflation is usually performed at low pressures (<6 atm) for 10–20 s. Typically, 2–5 segments are treated per session to minimize reperfusion injury, with additional sessions scheduled at 2–6-week intervals until hemodynamic targets are achieved.

## 8. Complications and Their Management

Complications of BPA have declined significantly but remain a principal concern. RPI, characterized by pulmonary edema and hypoxemia in reperfused segments, is the most common serious complication. In early series, RPI incidence was as high as 60%, but modern techniques have reduced this to 5–20% [21,22,24,28,49,50]. RPI typically presents within 24 h of the procedure and is often self-limited; management includes supplemental oxygen, diuretics and non-invasive ventilation. Severe cases may require mechanical ventilation or extracorporeal membrane oxygenation. Pre-treatment with riociguat and staging procedures to limit the number of segments treated at each session appear to reduce RPI risk [49,51]. Pulmonary artery injury (dissection or perforation) presents with hemoptysis or sudden hemodynamic deterioration. Minor perforations are often managed with balloon tamponade and reversal of anticoagulation. Major injuries may necessitate covered stent placement or coil or gelatin sponge embolization; Ejiri et al. reported a 98% success rate with gelatin sponge or covered stent treatment and no difference in long-term survival compared with uncomplicated cases [52]. Arrhythmias such as atrial fibrillation and heart block, vasovagal reactions, contrast-induced nephropathy and radiation exposure are additional considerations.

## 9. Clinical Outcomes: Randomized Trials

The modern era of BPA has been shaped by a few randomized trials (Table 2). The RACE trial randomized 105 patients to BPA or riociguat [51]. BPA produced a 60.1% reduction in PVR versus 33.3% with riociguat and resulted in larger improvements in mPAP, NT-Pro BNP and WHO functional class. Serious adverse events occurred more frequently in the BPA group, particularly hemoptysis and lung injury; however, no procedure-related deaths or treatment discontinuations due to adverse events were reported. A post hoc analysis of the RACE trial demonstrated that both BPA and riociguat effectively reduce RV afterload in patients with CTEPH; however, BPA achieved a greater decrease in RV afterload, and improvement in RV function was observed only with BPA [53]. The MR BPA trial in Japan randomized 61 patients with inoperable CTEPH to BPA or riociguat; after one year, BPA resulted in greater reductions in mPAP (−16.3 mmHg versus −7.0 mmHg) and greater improvement in PVR and quality of life scores [54]. Recently, the THERAPY-HYBRID-BPA trial investigated whether continuing riociguat after successful BPA provides additional benefit [55]. The continuation of riociguat following BPA mitigated the deterioration of exercise tolerance without increasing adverse events. These findings support the use of riociguat as a complementary treatment strategy for inoperable CTEPH after BPA. Together, these randomized trials establish BPA as a valuable modality for hemodynamic improvement and highlight the complementary role of medical therapy.

## 10. Clinical Outcomes: Registry and Observational Studies

Multiple prospective and retrospective registries provide real-world evidence for BPA outcomes (Table 3). The international multicenter prospective BPA registry led by Lang analyzed 484 patients from 18 centers across Europe, Japan and the United States [28]. Staged BPA reduced the median mPAP from 43 to 25 mmHg and the median PVR from 619 to 244 dyn·s·cm^−5^, accompanied by significant improvements in cardiac index and 6MWD. BPA-related complications occurred in 11.3% of sessions, with thoracic events—including lung injury and hemoptysis—reported in 9%. Notably, in-hospital mortality was 0%. Predictors of a greater reduction in PVR included higher baseline mPAP, opening five or more lesions, and treatment at experienced BPA centers. At 3-year follow-up, overall survival from the first BPA session was 94.1%, with no significant difference in patients with prior PEA.

The worldwide CTEPH Registry reported long-term outcomes for 1009 patients treated with PEA (60%), BPA (18%) or medical therapy across 34 centers in 20 countries [56]. Three-year survival was 94% after PEA, 92% after BPA and 71% with medical therapy alone. The registry demonstrated that advanced New York Heart Association functional class (IV versus I/II), elevated PVR at diagnosis, higher right atrial pressure, and increased follow-up mPAP were independent predictors of pulmonary hypertension–related mortality, irrespective of treatment strategy. These findings underscore that, with comprehensive multidisciplinary evaluation and a multimodal therapeutic approach incorporating PEA, BPA, and medical therapy, excellent outcomes can be achieved, reflected by an overall 3-year survival rate of 89%. The Polish multicenter registry, which included 236 patients undergoing BPA, demonstrated a 15-mmHg reduction in mPAP, an approximate 50% decrease in PVR, and a significant improvement in 6MWD from 341 ± 129 to 423 ± 136 m (*p* < 0.001). RPI occurred in 6.4% of sessions, and the 3-year survival rate was 92.4% [57].

Observational data have further clarified the role of BPA in specific clinical settings. In a U.S. cohort, Bashir et al. analyzed 211 BPA sessions performed in 77 patients using a refined technique incorporating pressure-wire guidance and the use of undersized balloons during initial sessions [49]. The study demonstrated a 26% reduction in PVR (6.5 ± 3.4 WU to 4.8 ± 2.9 WU, *p* < 0.001), a 71.7 m improvement in 6MWD, and a hemoptysis rate of 4.7%. In this study, preprocedural use of riociguat, high number of BPA sessions, and lower baseline pulmonary artery compliance were associated with favorable hemodynamic and/or functional response after BPA. Masaki et al. analyzed the CTEPH AC registry, a Japanese nationwide CTEPH registry, comparing BPA and PEA; they found similar overall mortality between the two modalities but greater improvement in renal function with BPA [58]. Gerges et al. examined left ventricular filling pressures in CTEPH and found that elevated left ventricular filling pressure (defined as mean pulmonary arterial wedge pressure or left ventricular end-diastolic pressure > 15 mmHg) occurs in about 10.6% of patients and is linked to reduced survival, indicating an additional disease component within CTEPH that independently influences prognosis [27]. Collectively, registry data affirm that BPA provides durable hemodynamic and functional benefits with improving safety as experience accumulates.

Across major registries and observational studies, follow-up after BPA is typically structured around several key time points. Early reassessment is commonly performed within 1–3 months after completion of staged BPA sessions, focusing on clinical status, biomarkers, echocardiography, and, in selected cases, right-heart catheterization [41,56]. Functional and hemodynamic improvements are frequently documented by 6 months, with many cohorts reporting sustained reductions in PVR and improvements in exercise capacity [25,59,60]. A 12-month follow-up is routinely included in observational studies to evaluate the durability of BPA-related benefits, while longer-term outcomes are assessed through annual follow-up in large registries, incorporating functional class, imaging-based perfusion assessment, and survival analysis [44,56,61]. These follow-up patterns reflect real-world clinical practice and provide the framework through which long-term outcomes after BPA have been evaluated.

## 11. Comparison with Pulmonary Endarterectomy and Medical Therapy

PEA remains the definitive treatment for operable CTEPH, with reported perioperative mortality of <5% and long-term survival exceeding 90% at 3 years [56]. However, PEA is technically demanding and available in limited centers [18,62]. Patients with distal disease, significant comorbidities or advanced age may not be candidates. BPA offers an alternative for these patients, restoring perfusion to distal segments and improving hemodynamics. Contemporary registries suggest that BPA provides long-term survival comparable to PEA, while offering the advantages of a less invasive strategy, including shorter individual hospital stays and lower peri-procedural morbidity [56,58,61,63,64]. Patients treated with BPA typically require multiple staged sessions, whereas PEA is often a single procedure. PEA may provide more complete relief of mechanical obstruction in proximal disease, while BPA is better suited for distal webs and bands. The 2022 ESC/ERS guidelines assign BPA a relatively modest recommendation (Class IIb) in technically operable patients whose lesions are predominantly distal and for whom the overall balance of benefit and risk makes PEA less favorable [19]. In response, a prospective randomized study, the GO-CTEPH trial (NCT05110066), is in progress to directly compare PEA with BPA in patients deemed suitable for either approach based on multidisciplinary evaluation of distal disease distribution [65].

Medical therapy remains important for both operable and inoperable patients, especially for those with microvascular disease. Riociguat improves hemodynamics and functional capacity and may reduce RPI when used before BPA [12,51]. Future studies are expected to explore combination strategies using endothelin receptor antagonists and prostacyclin analogs in conjunction with BPA.

## 12. Special Populations and Subgroups

Elderly patients constitute an increasing proportion of the BPA population. Shinya et al. evaluated 157 patients with CTEPH, categorizing them into an older group (≥75 years, *n* = 39) and a younger group (<75 years, *n* = 118) [66]. They found that BPA produced hemodynamic and functional improvements in the older group that were comparable to those observed in the younger group, with an acceptable safety profile. Although overall mortality was higher in the older group, all deaths were attributable to non–pulmonary hypertension causes, indicating that this difference did not affect the interpretation of BPA-related outcomes. Sex-related differences in outcomes have been investigated, and a recent study reported that female patients experienced slightly less functional improvement while exhibiting similar complication rates [67]. Patients with coexisting left-heart disease pose unique challenges; elevated left-ventricular filling pressure increases risk for pulmonary edema during BPA. Gerges et al. found that 10.6% of CTEPH patients had elevated left-heart filling pressure, which independently predicted mortality [27]. In such patients, careful fluid management, pre-procedural optimization and possibly smaller balloons or fewer segments per session are prudent. Patients with CTEPD may benefit from BPA, particularly when symptoms are severe; early data suggest improvements in exercise capacity, but long-term hemodynamic benefit is uncertain and further studies are needed [60]. Patients with congenital heart disease, myeloproliferative disorders or antiphospholipid syndrome may have unusual thrombotic patterns requiring individualized strategies [68].

## 13. Guidelines and Consensus Statements (Table 4)

The 2022 ESC/ERS guidelines assign a Class I, Level B recommendation for BPA in patients with inoperable CTEPH or persistent pulmonary hypertension after PEA, emphasizing referral to specialized centers with multidisciplinary teams [19]. The guidelines recognize that evidence is based on non-randomized studies and few randomized trials but highlight the substantial hemodynamic benefits and improving safety record. The Japanese Circulation Society issued comprehensive procedural indications and recommendations, including the use of small balloons, low inflation pressures, and staged sessions, and advised prioritizing lesions with favorable morphological characteristics [69]. The AHA/ACC scientific statement underscores that BPA should be considered as part of an integrated treatment plan with PEA and medical therapy and calls for prospective registries and randomized controlled trials to standardize definitions of success and complications [70]. The ESC working group consensus statement offers practical guidance on patient selection and preparation, imaging, technical details, complication management and follow-up [29]. These documents converge on the need for high-volume centers, multidisciplinary teams and individualized treatment strategies.

**Table 4:** Guideline and Consensus Recommendations on BPA.

Guideline/Statement	Recommendation Level	Indications	Notes
ESC/ERS Guidelines 2022	Class I, Level B	Inoperable CTEPH; persistent PH after PEA	Referral to specialized centers; staged BPA; emphasizes multidisciplinary teams
Japanese Circulation Society 2019	Class I, Level C	Inoperable CTEPH	Use small balloons and low inflation pressures; staged sessions
AHA/ACC Scientific Statement 2024	Scientific statement	Integrate BPA with PEA and medical therapy	Calls for prospective registries and randomized trials
ESC Working Group Consensus 2023	Consensus statement	Symptomatic CTEPH, including patients who declined PEA	Provides practical guidance on patient selection, imaging, procedure and follow-up

Abbreviations: BPA: balloon pulmonary angioplasty; CTEPH: chronic thromboembolic pulmonary hypertension; PH: pulmonary hypertension; PEA: pulmonary endarterectomy.

## 14. Future Directions

Numerous questions remain regarding the optimal use of BPA. Ongoing trials are exploring the sequencing of BPA and medical therapy, including whether pre-treatment with riociguat or other agents reduces complications and whether continuation after BPA confers additional benefit. Dedicated devices such as scoring balloons, cutting balloons and intravascular lithotripsy are being tested to address more calcified or resistant lesions. The role of BPA in CTEPD and in patients with concomitant left-heart disease or chronic kidney disease needs further study. Artificial intelligence and machine-learning algorithms are poised to transform imaging interpretation, lesion classification and procedural planning. Combined approaches with PEA, such as hybrid procedures where proximal lesions are surgically removed and distal segments treated percutaneously, may expand the treatable population. Finally, addressing disparities in the diagnosis and management of pulmonary embolism and CTEPH is critical. The scientific statement from the AHA highlights significant differences in incidence, treatment and outcomes based on race, sex and socioeconomic status and calls for targeted strategies to ensure equitable access to care [71].

## 15. Conclusions

BPA has evolved from an experimental intervention to a cornerstone of therapy for patients with inoperable or residual CTEPH. Randomized trials and observational studies consistently demonstrate that BPA markedly reduces PVR and mPAP, improves right-ventricular function and exercise capacity, and achieves survival rates approaching those of surgical treatment. Advances in imaging and procedural technique have reduced complications, particularly RPI and vascular injury, and have refined patient selection. Modern practice emphasizes multidisciplinary evaluation, staged procedures, use of small balloons, careful imaging guidance, and integration with targeted medical therapy. Guidelines from international societies endorse BPA as a Class I indication for inoperable CTEPH and stress the need for treatment at specialized centers. Future research will focus on optimizing sequencing with pharmacological therapy, developing new devices and imaging tools, expanding indications to special populations and ensuring equitable access. As evidence continues to accumulate, BPA is poised to play an increasingly central role in the personalized management of CTEPH.

## Figures and Tables

**Table 1:** Diagnostic modalities and high-risk features in CTEPH patients considered for BPA.

Modality	Main Information	High-Risk Features Relevant to BPA	Role in Follow-Up
Transthoracic echocardiography	RV size and function, TR velocity, estimated systolic PAP, pericardial effusion	Marked RV dilation; reduced RV longitudinal function (e.g., TAPSE < 17 mm); pericardial effusion; severe TR	Monitoring RV reverse remodeling; detecting recurrent pulmonary hypertension or pericardial effusion
V/Q scintigraphy	Extent and distribution of segmental perfusion defects	Large bilateral mismatched defects indicating extensive vascular obstruction	Qualitative assessment of perfusion recovery after BPA and screening for new perfusion defects
CT pulmonary angiography/DECT	Morphology of chronic thromboembolic lesions; RV/LV size ratio; lung perfusion blood volume	Extensive webs and bands in multiple lobes; mosaic attenuation; very low lung perfusion blood volume in several territories; type D/E lesions in segmental and subsegmental branches	Objective quantification of perfusion improvement and RV/LV remodeling; detection of new thromboembolic events
Selective pulmonary angiography	Detailed lesion type (A–E); segmental anatomy; collateral flow	Predominance of type D/E lesions; long occlusions; severe tortuosity; slow distal flow associated with higher risk of vessel injury and reperfusion edema	Confirmation of lesion response to previous BPA and guidance for repeat interventions
Right-heart catheterization	mPAP; PVR; RAP; cardiac output and index; mixed venous oxygen saturation	Markedly elevated PVR (>1000–1200 dyn·s·cm^−5^); RAP elevation; low cardiac index; low SvO_2_	Determining hemodynamic response to staged BPA
Cardiopulmonary exercise testing	Mechanism of exercise limitation; ventilatory efficiency; peak VO_2_	Very low peak VO_2_; steep VE/VCO_2_ slope; evidence of circulatory limitation despite preserved ventilatory mechanics	Objective assessment of functional recovery and prognostication after BPA

Abbreviations: CTEPH: chronic thromboembolic pulmonary hypertension; BPA: balloon pulmonary angioplasty; RV: right ventricle; TR: tricuspid regurgitation; PAP: pulmonary arterial pressure; TAPSE: tricuspid annular plane systolic excursion; V/Q: ventilation–perfusion; DECT: dual energy computed tomography; LV: left ventricle; PVR: pulmonary vascular resistance; RAP: right atrial pressure; SvO_2_: mixed venous oxygen saturation; VO_2_: volume of oxygen; VE/VCO_2_: Minute Ventilation/Carbon Dioxide Production.

**Table 2:** Landmark Randomized Controlled Trials of BPA.

Trial	Publication Year	Patients	Intervention	mPAP Change	PVR Change	Key Findings
RACE	2022	105	BPA vs. riociguat	−18.7 mmHg (BPA)	−60.1% (BPA)	BPA superior to riociguat; greater improvement in WHO functional class
MR BPA	2022	61	BPA vs. riociguat	−16.3 mmHg (BPA)	−59.6% (BPA)	BPA superior to riociguat; greater hemodynamic and functional improvement
				Peak cardiac index change	Adverse events	
THERAPY-HYBRID-BPA	2025	74	Riociguat continuation vs. discontinuation after BPA	−0·03 vs. −1.11 L/min per m^2^	No significant difference	Riociguat as adjunctive therapy after BPA

Abbreviations: BPA: balloon pulmonary angioplasty; mPAP: mean pulmonary arterial pressure; PVR: pulmonary vascular resistance.

**Table 3:** Outcomes of Registries and Observational Studies.

Study	Publication Year	Patients	mPAP Change	PVR Change	Complication Rate	Key Findings
International BPA registry	2025	484	−15 mmHg	−57%	11.3%	Durable hemodynamic improvement; 3-year survival 94.1%
Worldwide CTEPH registry	2024	1009	−18 mmHg	−59%	12%	3-year survival: 94% for PEA, 92% for BPA, 71% for medical therapy
Polish multicenter registry	2022	236	−15 mmHg	−50%	9%	Improved 6MWD and functional class
U.S. refined BPA cohort	2023	77	−6.4 mmHg	−26%	1.4% of major complications	Favorable factors: pre-BPA riociguat, high number of BPA sessions, and lower baseline PA compliance
Japanese nationwide CTEPH registry	2024	369	−19.9 mmHg	−60%	Not available	Similar mortality between BPA and PEA; renal function improved more with BPA

Abbreviations: mPAP: mean pulmonary arterial pressure; PVR: pulmonary vascular resistance; BPA: balloon pulmonary angioplasty; CTEPH: chronic thromboembolic pulmonary hypertension; PEA: pulmonary endarterectomy; 6MWD: 6 min walk distance.

## Data Availability

No new data were created or analyzed in this study.
